# 4190 White Matter Integrity in Hemodialysis Patients

**DOI:** 10.1017/cts.2020.321

**Published:** 2020-07-29

**Authors:** Wesley Richerson, Dawn Wolfgram, Brian Schmit

**Affiliations:** 1 Medical College of Wisconsin; 2 Medical College of Wisconsin Department of Medicine; 3 Marquette University Biomedical Engineering

## Abstract

OBJECTIVES/GOALS: The purpose of this study is to understand how hemodynamics during dialysis in End-Stage Renal Disease (ESRD) patients on hemodialysis (HD) affect white matter health and how those effects cause cognitive impairments. METHODS/STUDY POPULATION: We collected demographic data, comorbidities, intradialytic measurements of blood pressure and cerebral oximetry, cognitive measures in several domains using NIH Toolbox Cognition Battery, diffusion-weighted and anatomical MRIs for 20 participants on HD. Specific tracts were identified using tractography and were used to calculate the average DTI measurements in each tract. Regression analysis was used to examine the relationship between mean DTI measurements of white matter integrity and cognitive performance scores. In addition, we compared diffusion MRI and T1 anatomical images of 16 healthy age-matched controls from a previous study. RESULTS/ANTICIPATED RESULTS: In our cohort 18 participants had imaging data that could be used in the analysis. We found widespread decreases in DTI white matter integrity compared to healthy age-matched controls, mean whole-brain fractional anisotropy was .3218 in the HD cohort and .3472 in controls p = .0018. Decreased integrity was found in most of the tracts identified but more decreased in tracts implicated in cognition. Partial regression analysis identified significant relationships between the white matter integrity of the left superior longitudinal fasciculus and overall fluid cognitive performance, R = .5525, p = .0174 before multiple comparisons correction when controlling for differences due to age. DISCUSSION/SIGNIFICANCE OF IMPACT: We found a widespread decrease in white matter integrity and significant correlations between cognitive performance and specific tract integrity in our HD cohort using regions identified by tractography imaging analysis. This analysis shows that HD patients have decreased white matter health and identifies several tracts that are important for cognitive performance in HD patients.

